# Migraine Among University Students: Prevalence, Characteristics, and Sociodemographic Influences

**DOI:** 10.3390/healthcare13141746

**Published:** 2025-07-18

**Authors:** Maria Axiotidou, Hariklia Proios, Theodoros Karapanayiotides, Doxa Papakonstantinou

**Affiliations:** 1 Department of Educational and Social Policy, University of Macedonia, 156 Egnatia Street, 54636 Thessaloniki, Greece; maxiotidou@uom.edu.gr (M.A.); hproios@uom.gr (H.P.); 2 2nd Department of Neurology, AHEPA University Hospital, School of Medicine, Aristotle University of Thessaloniki, 1 S.Kyriakidi Street, 54636 Thessaloniki, Greece; tkarapanayiotides@auth.gr

**Keywords:** migraine, prevalence, university students, sociodemographic influences, Greece

## Abstract

**Background**: Migraine is a highly prevalent and disabling neurological disorder among university students that has significant impacts on personal and socioeconomic levels. Despite its impact, migraine remains underdiagnosed and undertreated. **Objective**: This study aimed to estimate the prevalence of probable migraine among university students in Greece and explore its association with sociodemographic data. **Methods**: A cross-sectional, questionnaire-based study was conducted between September 2023 and January 2024 among university students in Greece using a convenience sampling method. The Headache Screening Questionnaire—English Version (HSQ-EV) was used to screen for probable migraine, along with additional questions assessing demographic characteristics. Descriptive statistics and bivariate analyses were performed. **Results**: The prevalence of probable migraine was 20%. Female students were more likely to experience migraine compared to males. Migraine was also statistically significantly associated with marital status and employment status. In a multivariate logistic regression model including sex assigned at birth, age, educational level, marital status, and employment status, older age was independently associated with higher odds of migraine. **Conclusions**: Migraine is a prevalent health issue among university students in Greece, with clear gender and sociodemographic associations. Future studies with larger, more representative sample sizes and the use of validated diagnostic tools are needed to understand its determinants and inform targeted interventions.

## 1. Introduction

Migraine ranks among the top 10 diseases with the highest prevalence [1] and is recognized as the second leading cause of disability worldwide [2], affecting more than 1 billion people globally [3]. According to the Global Burden of Disease Study 2019, the estimated prevalence of migraine was approximately 581,761,847.2 cases, representing a 16% increase since 1990 [4]. Migraine prevalence peaks among individuals aged 30–34 [5]. Despite its high prevalence, migraine remains under-recognized and under-treated even in affluent countries [6,7], likely due to misconceptions about the condition and a lack of awareness among healthcare providers and the general population.

Migraine is a neurological disorder characterized by recurrent, pulsating moderate-to-severe headache episodes [8], often accompanied by neurological symptoms such as photophobia, phonophobia, nausea or vomiting, and reduced mobility [9]. It can be categorized as episodic migraine (EM; <15 headache days/month averaged over the last three months) and chronic migraine (CM; ≥15 headache days/month averaged over the previous three months) [10]. Approximately one-third of individuals with migraine experience transient neurological symptoms known as aura [11]. These disturbances can vary widely in form and severity, ranging from visual disturbances—such as flashing lights, zigzag patterns, or loss of the visual field—to other sensory or speech-related disruptions [12]. Aura often occurs before the onset of the headache but can also accompany the migraine episode itself [13].

This chronic condition not only causes significant disability, but also imposes a considerable and multifaceted burden on both individuals and society [14]. Migraine negatively affects health-related quality of life [15], confirming that individuals with migraine experience substantial difficulties in both physical and mental health [16]. Migraine patients consistently report a significant impact on familial and social relationships [14], as well as financial well-being [16]. Additionally, migraines are associated with reduced work productivity due to activity impairment [17], leading to approximately 250 million lost days from work or school annually [18].

Migraine is also a prevalent health concern among university students [6]. Existing literature indicates an increasing trend of migraine among this population [19]. Migraine prevalence rates among university students vary widely [20]. A study by Rustom et al. in the United Arab Emirates reported a high migraine prevalence of 26.3%, with almost 60% of university students experiencing moderate-to-severe disability and about 30% requiring hospital admission [6]. Conversely, other studies reported lower prevalence rates of 6.57% and 7.91% among medical students in China [21,22]. The incidence rate was higher among lower-grade university students [21,22] and female students [20].

Migraine significantly impacts the quality of life of university students [19], as many report increased levels of stress, depression, anxiety, and irregular sleep as consequences of migraine [19,23,24]. Stress during studying and exams, irregular sleep, and noise are among the most frequent triggers [25]. Several studies reported that migraine is associated with low academic achievement [19,26] and limited daily functioning [19,27]. Nevertheless, the majority of university students continue to attend educational activities despite headache attacks [19]. However, despite this growing international evidence, relevant epidemiological studies from Greece remain limited. Given the methodological challenges of clinical confirmation in large student populations, most relevant studies—including the present one—relied on self-reported screening instruments to estimate migraine prevalence. While these tools enable practical and large-scale assessment, they do not substitute for formal diagnosis by a clinician. As such, the identification of migraine cases in this context should be considered probabilistic and interpreted with appropriate caution.

While many studies evaluated the prevalence of migraine among university students globally, there is a scarcity of epidemiological data from Greece. Most research on migraine among university students focused on general prevalence rates and has not examined whether sociodemographic factors, such as employment and marital status, may influence these rates. Furthermore, previous studies generally targeted specific academic fields or geographical regions, leaving a gap in understanding how these variables interact with migraine prevalence across various university populations.

The objective of the current study is to address this gap by examining migraine prevalence among university students in Greece, with a specific focus on how various sociodemographic parameters, such as age, gender, employment status, marital status, and educational level, may affect migraine prevalence. The study also explores headache characteristics, symptom profiles, and other contextual variables that are relevant to the migraine burden in this population. By concentrating on an underrepresented group in the existing literature and considering factors such as employment status, this study aims to provide new insights into how social and individual factors influence migraine prevalence and severity. This research contributes to the growing body of knowledge regarding the challenges posed by migraine for university students, offering valuable information that could inform preventive strategies and support programs tailored to the needs of Greek university students.

## 2. Materials and Methods

### 2.1. Study Population

A cross-sectional, questionnaire-based study was conducted during the 2023–2024 academic year. The recruitment period commenced on 1 September 2023 and concluded on 31 January 2024, targeting university students aged 18 and above in Greece. Participants included university students from a variety of fields and academic years, encompassing both undergraduate and postgraduate levels (master’s and doctoral students), ensuring a diverse and representative sample. The participants varied in age, gender, and nationality, with an observed age range extending up to 56 years, reflecting students pursuing a second degree or beginning their studies at an older age. University staff members were excluded from the research sample.

In this study, employment status refers to students’ part-time or full-time work during their studies. This variable was included due to the higher levels of employment among university students in Greece, which can impact health outcomes, such as migraine. Specifically, working university students may experience increased levels of stress and irregular sleep—factors that commonly trigger migraine episodes. The principal inclusion criterion for participant selection was being a current university student across all academic levels during the study period. Potential respondents were excluded if they had secondary headaches, were not enrolled in any university, were part of the university faculty, were unwilling to participate, or abandoned their studies during the study period. This approach aimed to comprehensively represent the university student population, facilitating the potential generalization of the findings to the broader university community.

The study was conducted in three universities throughout Greece, representing a range of geographic and academic diversity, including large metropolitan institutions. Students from various disciplines and academic years were eligible to participate in order to include as many students as possible. A convenience sampling process was employed.

### 2.2. Data Collection Instruments

The data collection process consisted of two sections. The first section gathered sociodemographic and personal data, such as sex, age, marital status, educational level, employment status, and place of residence. The second section comprised a questionnaire related to headache. This section focused on migraine prevalence, with data collected through the Headache Screening Questionnaire—English Version (HSQ-EV), a brief screening tool designed to assess the presence of headache types, including migraine and probable migraine [28]. The average completion time was estimated to be around 7 min. A total of 565 participants completed the questionnaire and were included in the analysis.

The HSQ-EV was developed and validated in the Netherlands [28]. It comprises 10 items that reflect four diagnostic domains derived from the International Classification of Headache Disorders, 3rd edition beta version (ICHD-3 beta): (A) frequency of attacks, (B) duration of headache, (C) pain characteristics, and (D) associated symptoms (such as nausea or photophobia). Although the HSQ-EV incorporates migraine-specific criteria and is used to identify probable migraine, it is not a migraine-specific tool. Due to its general focus on headache-related symptoms, it may also capture tension-type headaches, which are also classified as primary headaches.

The HSQ-EV yields a total score ranging from 0 to 8, with higher scores indicating greater alignment with migraine symptom criteria. According to the validation study by Van der Meer et al. [28], a score of 8 corresponds to a migraine diagnosis, fulfilling all four ICHD-3 beta diagnostic domains, with a sensitivity of 0.69 and a specificity of 0.90 compared to a clinical ICHD-3 beta diagnosis. A score of ≥6 (e.g., 6 or 7) corresponds to probable migraine, indicating that the participant meets three of the four required criteria, consistent with the ICHD-3 beta definition of probable migraine. For this category, sensitivity increases to 0.89, although specificity decreases to 0.54.

For the purpose of our analysis, migraine presence was operationally defined as an HSQ-EV score of 6 or higher, capturing both probable and definite migraine cases. This grouping was necessary due to the small number of participants with a score of 8 (*n* = 23) and reflects the continuum of migraine symptom severity captured by the tool.

We acknowledge that this approach may slightly overestimate migraine prevalence, particularly due to the moderate specificity of the HSQ-EV in identifying probable migraine cases. Nevertheless, this strategy aligns with the intended screening function of the HSQ-EV. It is important to emphasize that the HSQ-EV is a screening instrument, not a diagnostic tool, and clinical confirmation by a neurologist was not feasible within the current study design. Moreover, the HSQ-EV is based on the beta version of the ICHD-3 and is not specific to migraine, which may lead to misclassification, particularly between migraine and tension-type headaches. Additionally, the absence of a clinical diagnosis by a neurologist restricts diagnostic precision.

The cross-sectional adaptation of the HSQ-EV into Greek followed international guidelines for the translation and cultural adaptation of health-related questionnaires (e.g., WHO protocols). The HSQ-EV was translated into Greek using a standard forward–backward translation process. First, a bilingual researcher translated the original English version into Greek. An independent translator, who was unaware of the original version, then back-translated the Greek version into English. The two versions were compared, and any discrepancies were resolved through discussion and iterative revisions until consensus was reached on the accuracy of the translation. This process ensured that the Greek version of the questionnaire retained both linguistic and conceptual equivalence to the original. Expert feedback was incorporated to ensure that the translated items were culturally appropriate and easily understood by the target population. To further ensure content validity, a bilingual healthcare professional reviewed the translation, and their feedback was integrated into the final version.

A pilot study was conducted with 10 university students to evaluate the clarity and cultural relevance of the translated items. Participant feedback led to minor wording adjustments to enhance comprehensibility, and no significant difficulties were reported in understanding the questions. All participants provided informed consent before participation, and responses were collected anonymously to ensure confidentiality.

Due to the structure of the HSQ-EV, comprising non-scale items, it was not possible to calculate Cronbach’s alpha for the Greek version. Nevertheless, the thorough translation process, pilot testing, and expert consultation provide confidence in the internal consistency and overall appropriateness of the Greek version for use in this context.

To the best of our knowledge, no previous validation of the HSQ-EV has been conducted among the Greek population. Therefore, this study represents the first attempt to apply the instrument in a Greek-speaking context. Although a full psychometric validation was not feasible due to the tool’s format, we followed accurate translation procedures, expert judgment, and pilot testing to ensure the cultural and linguistic adequacy of the translation. While this study marks the first known application of the HSQ-EV in a Greek-speaking population, a comprehensive psychometric validation (e.g., test–retest reliability, internal consistency, and construct validity) was not performed. This decision was based on the instrument’s design as a categorical screening tool aligned with diagnostic criteria, rather than as a scale intended to measure underlying latent constructs. Consequently, conventional psychometric analyses, such as Cronbach’s alpha or factor analysis, are not applicable. Moreover, the exploratory, cross-sectional nature of the study did not permit an assessment of test–retest reliability or clinical diagnostic comparison. Nonetheless, the translation and cultural adaptation process adhered to international best practices, including forward–backward translation, expert review, and pilot testing, which support the instrument’s content and conceptual equivalence in the Greek context.

### 2.3. Data Collection

This research initiative adhered to the principles of the Helsinki Declaration of Biomedical Ethics. Additionally, formal approval was obtained from the Ethics Committee of the University of Macedonia in Greece before the commencement of the study (Ref No: 6/24-11-2021). This strict adherence to ethical standards ensures the integrity and reliability of the study’s findings.

The convenience sampling method was employed to recruit participants, targeting a specific university student population that researchers could easily approach. Participants were recruited using a mixed-method approach. For online recruitment, the questionnaire link was distributed through digital networks and social media, allowing the survey to reach a diverse array of academic groups. In-person recruitment occurred during scheduled university lectures, where printed copies of the questionnaire were distributed in collaboration with academic faculty. This complementary dual approach was undertaken to enhance survey accessibility and maximize participation across the selected institutions.

Specifically, the survey was developed and conducted using the digital platform Google Forms. Before data collection, each participant was thoroughly informed about the research procedures and objectives through an introductory page on the online questionnaire platform. Informed consent was obtained electronically from online participants. The researchers distributed the questionnaire link to participants’ email addresses after collecting their information regarding the study. Participants could also complete the questionnaire online if they had access to social media. Initially, they were required to read the detailed consent form and indicate their agreement to participate by selecting a checkbox before proceeding. This ensured the voluntary nature of participation, as well as anonymity and the confidentiality of the gathered data. Contact information was provided on the initial page of the survey for participants who had questions regarding the study.

For in-person participation, informed consent was obtained verbally by the researchers and documented with a signature on a printed consent form. Researchers also informed participants of their right to withdraw from the study at any time and assured them that all data would be kept private. The signed consent forms were securely stored to ensure confidentiality. For online participants, informed consent was obtained electronically via a mandatory checkbox at the beginning of the questionnaire, following a detailed information page outlining the study’s purpose, voluntary nature, and data privacy measures.

The questionnaire was distributed both online (via Google Forms) and in person using printed versions handed out during university classes in collaboration with academic staff. This dual approach aimed to enhance inclusivity and representativeness among university students. A total of 565 students participated in the study, with no incomplete or inconsistent responses. Although the exact number of students who received or accessed the survey invitation is unknown, it is estimated that approximately 1000 individuals from various universities were invited, resulting in a response rate of about 56.5%.

No formal sample size calculation was performed prior to data collection. Given the descriptive, exploratory, and cross-sectional nature of the study, as well as the use of convenience sampling, precise power estimation was not feasible. Instead, the goal was to recruit the largest feasible number of participants during the study period to enhance statistical reliability. While no formal sampling quotas were applied, efforts were made to ensure diversity by disseminating the questionnaire across academic levels and fields of study. Nonetheless, the absence of an a priori sample size justification may limit the ability to detect small effect sizes and should be considered when interpreting non-significant findings.

### 2.4. Statistical Analysis

The present study assessed the prevalence of migraine among university students by analyzing the collected data. Data were coded and entered using Microsoft Office Excel 2010, and all statistical analyses were conducted using IBM Statistical Package for the Social Sciences (SPSS) for Windows, version 20.0. Simple descriptive statistics were used to summarize the numerical variables of the sample, as well as the frequency and percentage of categorical variables. Means and standard deviations (SD) are reported for normally distributed continuous variables. However, since the Shapiro–Wilk test indicated a non-normal distribution, non-parametric methods were used for continuous data, and the median and interquartile range (IQR) are presented instead. Categorical variables are reported as absolute and relative frequencies, and summary statistics for continuous variables are tabulated. The normality of continuous variables was assessed using the Shapiro–Wilk test. A *p*-value < 0.05 was considered statistically significant. To evaluate the association between demographic characteristics and migraine scores (as measured by the HSQ-EV), a non-parametric statistical analysis tool, the Mann–Whitney U test, was used. Pearson’s chi-squared test was applied to examine the relationship between demographic characteristics and the presence of migraine. Associations between covariates of interest and migraine prevalence were further evaluated using binary logistic regression analysis. A multivariate logistic regression model was constructed to estimate adjusted odds ratios (ORs) along with corresponding 95% confidence intervals (CIs) and *p*-values. Variables included in the multivariate model were selected based on their clinical relevance and prior evidence. The multivariate logistic regression model included the following covariates: sex, age (dichotomized at 21 years), marital status (married vs. unmarried/divorced), educational level (undergraduate vs. postgraduate), and employment status (employed vs. unemployed). These variables were selected based on their clinical relevance and statistically suggestive results from the univariate analyses. The age cut-off at 21 years was based on the median age of the sample to create two approximately balanced groups and avoid sparsity in either category, thus improving model stability. This approach is commonly used when no established or theoretically justified threshold exists. Preliminary findings from univariate analyses were used for exploratory purposes to identify potential associations between demographic characteristics and migraine outcomes. However, the primary conclusions were based on a multivariate binary logistic regression model, which allowed for the adjustment of potential confounding variables and the identification of independent factors associated with the presence of probable migraine.

## 3. Results

A total of 565 participants completed the questionnaire between September 2023 and January 2024. The sample consisted predominantly of females (82.7%), with a median age of 21 years (IQR: 19–25). Most participants were unmarried (90.1%), and the majority were undergraduate students (63.8%). In terms of employment status, 63.9% were unemployed, while 36.1% were employed across various sectors, including the public and private sectors, or were self-employed (Table 1).

As shown in Supplementary Table S1, 42.7% of participants reported experiencing headaches ≥10 times in their lifetime, while 67.3% reported headache episodes occurring 0–4 times. Regarding headache frequency, approximately 50% of the participants (49.9%) reported experiencing headaches ≥1–<15 times per month, with only a small percentage experiencing headaches ≥15 times per month. More than half of the students (55.8%) reported headache durations without medication ranging from 30 min to 4 h. In terms of headache quality, most participants (62.7%) described their headaches as tight or pressing, followed by a pulsating sensation (19.6%) and a burning or stabbing sensation (6.5%). Compared with other studies, a slight majority (51.7%) in our study reported bilateral pain, while 48.3% reported unilateral pain. The majority of participants (93.1%) rated their pain intensity as mild to moderate, and 46% of participants reported avoiding their daily activities during headache episodes. Phonophobia was the most common symptom associated with headache attacks (53.6%), followed by photophobia (39.6%) and nausea and/or vomiting (24.8%).

Preliminary univariate analyses were conducted to explore the crude associations between demographic variables and migraine severity (as measured by score) or presence (as indicated by probable migraine) (Table 1). A statistically significant difference was found in migraine scores between males and females (*p* = 0.0004), with females reporting higher scores, suggesting that women may be more severely affected by migraines than men. Although effect size measures (such as rank–biserial correlation) were not calculated, the observed difference in migraine scores between sexes (*p* = 0.0004) aligns with well-documented sex differences in migraine prevalence and severity reported in the literature. This consistency reinforces the practical relevance of the finding beyond its statistical significance. No significant differences in migraine scores were found based on marital status, educational level, or employment status.

In the overall sample, the prevalence of probable migraine was 20%, with 113 individuals meeting the criteria for “probable migraine” based on their HSQ-EV scores. Among these, the majority were female (21% of the total sample) compared to males (15.3%) (Table 2). The median score was 4.0 (IQR: 3.0–5.0). According to the study design, participants with HSQ-EV scores of 6 or higher were classified as having probable migraine. This category includes both probable and definite migraine classifications to account for the self-reported nature of the data and the absence of clinical diagnosis.

Pearson’s chi-squared tests indicated that the presence of probable migraine was statistically significantly associated with marital status (*p* = 0.020) and employment status (*p* = 0.044) (Table 3). Specifically, married participants exhibited a higher prevalence of migraine (33.31%) compared to unmarried or divorced participants (18.8%). Similarly, employed participants had a higher prevalence of migraine (24.5%) compared to unemployed participants (17.5%). No significant difference was found between males and females in migraine prevalence using chi-squared testing (*p* = 0.201), despite differences in migraine severity scores. Additionally, no significant difference was observed between postgraduate and undergraduate students in terms of migraine scores (*p* = 0.161).

Multivariate binary logistic regression analysis of factors related to the presence of migraine is presented in Table 4. Among all factors examined, only participants aged ≥21 remained significantly associated with the presence of migraine [OR (95% CI) = 1.86 (1.09–3.16), *p* = 0.022], indicating that participants aged 21 or older had 86% higher odds of probable migraine compared to those under 21. Female participants showed 68% higher odds of migraine compared to males [OR (95% CI) = 1.68 (0.91–3.10), *p* = 0.100]; however, this association did not reach significance. Similarly, 29% higher odds of migraine were observed among undergraduate students compared to postgraduate students, without reaching significance [OR (95% CI) = 1.29 (0.73–2.26), *p* = 0.384]. Married participants had higher odds of migraine compared to unmarried or divorced individuals [OR (95% CI) = 1.59 (0.78–3.22), *p* = 0.202], and employed participants also had higher odds of migraine compared to unemployed individuals [OR (95% CI) = 1.26 (0.76–2.08), *p* = 0.374]. However, these associations did not achieve statistical significance.

These findings suggest that while some unadjusted associations (e.g., for marital and employment status) were statistically significant, they did not remain significant after adjusting for other variables. This highlights the importance of multivariate analysis in identifying independent predictors of migraine.

## 4. Discussion

This cross-sectional study estimated the prevalence of probable migraine among Greek university students and examined the influence of sociodemographic factors. Findings indicate a high prevalence of probable migraine, affecting 20% of the sample (15.3% of males and 21.0% of females). For analytical purposes, HSQ-EV scores of 6–8 were grouped into a single “probable migraine-positive” category due to the limited number of definite cases. This finding is consistent with several international studies [6,20,22,23,24,27,29,30,31].

The sample was disproportionately female, which may have contributed to an overestimation of the overall migraine prevalence. Sex was included as a covariate in the multivariate analysis to mitigate this imbalance; nonetheless, caution is warranted when generalizing these estimates to the broader student population.

Although females reported higher rates of probable migraine compared to males, this association was not statistically significant in the multivariate analysis. However, the observed trend aligns with well-established gender differences in migraine prevalence [6,19,20,21,24,25,27,32], while some studies reported no significant gender differences [23]. Further investigation is warranted in more demographically balanced samples.

Several biological and psychosocial mechanisms may explain the higher prevalence of migraine among women. Migraine is approximately three times more common in women, with frequency and severity changing across life stages due to hormonal fluctuations (e.g., menstruation, pregnancy, and menopause). Estrogen withdrawal is linked to migraine onset, while higher estrogen levels may be protective [33]. Additionally, behavioral and lifestyle factors, including higher stress, sleep disturbances, missed meals, and light sensitivity—all recognized migraine triggers [34,35]—may further increase female vulnerability. These findings support a multifactorial explanation of sex-based disparities in migraine prevalence that merits additional targeted study.

Univariate analyses showed significant associations between migraine prevalence and both marital and employment status, although these did not remain significant in the multivariate model. Married participants reported higher migraine prevalence than unmarried or divorced individuals (33.3% vs. 18.8%). While this may indicate a link between marital status and migraine risk, the clinical significance remains uncertain. The association may reflect underlying stress or lifestyle differences rather than a causal effect. Migraine has been linked to impaired interpersonal functioning and reduced relationship satisfaction [16], and reverse causality is also plausible—chronic migraine may contribute to relationship strain. Stress, increased social responsibilities, and lifestyle changes are common among married students and may heighten migraine vulnerability. However, evidence from other studies is mixed, with some reports showing no significant relationship between marital status and migraine [36]. Further research is needed to clarify whether relationship dynamics contribute to the onset or severity of migraine.

Employment status was also significantly associated with migraine. Employed students (part-time or full-time) reported a higher prevalence (24.5%) compared to unemployed individuals (17.5%). The challenge of balancing academic responsibilities with work-related stress may make employment a risk-enhancing factor. High levels of stress, long working hours, noise, and fatigue are identified as migraine triggers [37], which may explain the association. However, as with marital status, reverse causality must be considered. Migraine may impair job performance, reduce productivity, or hinder consistent employment. The bidirectional nature of this relationship complicates causal interpretation and underscores the need for longitudinal designs.

No statistically significant association emerged between migraine and educational level (undergraduate vs. postgraduate). This finding limits comparability with previous studies that reported heterogeneity by academic year or field [20,21,22,38]. Since the academic year was not analyzed, comparisons remain constrained.

When interpreting these associations, several sample-related limitations must be acknowledged. While participants’ place of residence was collected, all respondents attended urban universities, which limited geographic variation and prevented comparisons between rural and urban areas. Moreover, although employment status was recorded, other indicators of economic background, such as household or personal income, were not assessed. The majority of students were financially dependent on their families and lacked full-time employment, making it difficult to achieve meaningful economic stratification. Future studies should incorporate subjective measures of financial strain or parental socioeconomic status to examine their role in migraine prevalence more comprehensively.

Other studies in Greece, such as by Adamakidou et al. [39], reported even higher prevalence rates (e.g., 48% among nursing students), indicating substantial heterogeneity in the findings. Differences in academic field, exposure to stress, measurement instruments, and methodology may account for this variation. The high prevalence observed in this study may also reflect cultural and behavioral factors, such as academic stress, excessive screen time, disrupted sleep, and lifestyle changes, that are increasingly common among student populations. Importantly, although medical students (a high-risk group) were not included, prevalence remained elevated, suggesting that migraine is a broader issue across academic disciplines.

Data on migraine frequency, duration, and severity indicate that most students experienced low-frequency, mild-to-moderate episodes. Nearly half reported headaches less than once per month; only 2.3% experienced more than 15 per month. Most headaches lasted between 30 min and 4 h (55.8%), with 32.9% lasting under 30 min. While bilateral pain was more common (51.7%), this finding contrasts with prior studies, which characterized unilateral, pulsating headaches as more typical [24,40]. The severity of pain was generally mild (43.9%) or moderate (49.2%), with only 6.5% reporting severe pain [24,40]. Common accompanying symptoms included photophobia, phonophobia, nausea, vomiting, dizziness, neck pain, and cognitive difficulty [21,41]. Interestingly, most participants reported minimal functional interference, in contrast to other studies that emphasize migraine-related disability [41].

A number of methodological limitations must also be considered. First, migraine diagnoses were based solely on a self-administered screening tool (HSQ-EV) rather than clinical confirmation by a neurologist. Although the use of a self-administered questionnaire is common in epidemiological research, it introduces a degree of subjectivity and potential for misinterpretation of questions. Self-reported data may also lead to inaccuracies, as participants could underestimate their symptoms. The HSQ-EV, based on the ICHD-3 beta, lacks specificity and is not migraine-specific. Grouping probable and definite cases may have included individuals with other primary headache disorders, such as tension-type headaches, potentially inflating prevalence. Future studies should incorporate both self-reported screening tools and clinical validation, as well as the most current ICHD-3 criteria to improve diagnostic precision [30].

Second, the use of convenience sampling—without randomization or stratification—may have introduced bias. Participants were recruited from a limited number of departments and networks, and self-selection could have favored individuals with migraine-related experiences. This recruitment strategy was primarily chosen due to feasibility constraints and the absence of a centralized sampling frame for the national student population, which made probability-based sampling methods impractical.

The gender imbalance (82.7% female) likely influenced prevalence estimates, although statistical adjustments were made. Differences observed between males and females in migraine outcomes should therefore be interpreted with caution, as they may reflect sampling bias rather than true population-level disparities. Post-stratification weighting or sensitivity analyses were not conducted, which limits the generalizability to the broader student population. The study also lacked systematic data collection on participants’ academic field and year of study, an omission due to the descriptive nature of the research and the absence of prior hypotheses related to academic background.

Third, relevant behavioral and lifestyle variables—including screen time, sleep habits, physical activity, and body weight—were not measured. Although this decision was made to reduce respondent burden and avoid overly lengthy questionnaires, given the large sample and non-clinical setting, it constrained analytical depth. The inclusion of such variables in future research would enhance the understanding of potential modifiable risk factors. Additionally, migraine subtypes (e.g., with vs. without aura) were not classified, which may have provided a more nuanced view of migraine burden.

Univariate analyses were not adjusted for multiple comparisons, increasing the likelihood of false-positive findings. Caution is advised when interpreting preliminary associations that were not supported by multivariate analysis.

The timing of data collection (September to January) may also have influenced symptom reporting, as this period coincides with the academic workload associated with examinations and heightened stress. Seasonal effects could thus have contributed to the elevated frequency or severity of migraine episodes.

Although the HSQ-EV was back-translated by bilingual researchers, the translation process lacked formal linguistic validation or piloting in a demographically diverse sample. This may have affected the interpretability and consistency of responses. Future research should ensure rigorous cross-cultural adaptation and psychometric validation of measurement tools.

Despite these limitations, the study presents several strengths. The relatively large sample size (*n* = 565) provides sufficient statistical power for subgroup analyses. The inclusion of both undergraduate and postgraduate students enhances the breadth of applicability. The use of a validated screening instrument supports comparability with international studies and addresses a research gap by focusing on non-medical students, a population often underrepresented in migraine research.

Future studies should prioritize longitudinal designs, incorporate lifestyle and psychological variables (e.g., perceived stress, academic workload), and apply standardized tools, such as HIT-6 or MIDAS, to assess migraine-related disability and quality of life. Although these were omitted here to reduce participant fatigue, their inclusion would provide valuable insights into the functional impact of migraine in student populations. Furthermore, examining mediating variables, such as financial strain, coping strategies, and circadian disruption, would advance our understanding of the causal pathways. A multifactorial, interdisciplinary approach is essential to inform targeted prevention and support strategies for university students affected by migraine.

These limitations underscore the need for caution in drawing broad conclusions and highlight several methodological areas for improvement in future research.

## 5. Conclusions

This cross-sectional study highlights the significant prevalence of probable migraine among Greek university students, affecting 20% of participants, with a higher incidence observed among females. These findings are consistent with prior research, confirming that migraine is a common health issue in university populations worldwide and has substantial implications for students’ academic performance and overall quality of life.

Given the high prevalence, universities could implement health campaigns to raise awareness about migraine symptoms, triggers, and prevention strategies. Such initiatives may promote early detection and improve students’ ability to manage migraine effectively. Institutions might also consider offering targeted support services, such as flexible academic schedules, quiet study spaces, and access to healthcare professionals. Notably, the study identified a strong association between employment status and migraine prevalence. This suggests that universities could benefit from collaborating with employers to develop flexible working arrangements for students engaged in part-time work. Although marital and employment status were not retained as independent predictors in adjusted models, their significant univariate associations with migraine suggest that stress and lifestyle factors may contribute to migraine burden. These findings underscore the importance of considering students’ broader social and environmental contexts in migraine prevention and management strategies.

In conclusion, this study highlights the importance of universities recognizing and addressing the impact of migraine on student health, productivity, and academic performance. Through targeted interventions and ongoing research, institutions can better support students in managing migraine and enhancing their overall well-being. Tackling migraine in student populations is not only a matter of health promotion, but also a critical step toward fostering academic success, psychological resilience, and long-term professional readiness in higher education.

## Figures and Tables

**Table 1:** Demographic characteristics of the study population and corresponding median migraine scores (HSQ-EV) with *p*-values from the Mann–Whitney U test: total sample: *N* = 565.

	*n* (% of Total Sample)	Median Migraine Score (IQR)	*p*-Value
**Sex**			
Male	98 (17.3%)	3.0 (2.0, 4.0)	0.0004 *
Female	467 (82.7%)	4.0 (3.0, 5.0)	
**Age (in years)**			
Mean (SD)	23.56 (7.4)		
Median (Q25-Q75)	21 (19–25)		
n_miss_	1		
Shapiro–Wilk *p*-value *	<0.001		
**Marital status**			
Unmarried/divorced	520 (92%)	4.0 (3.0, 5.0)	0.094
Married	45 (8%)	4.0 (3.0, 6.0)	
**Educational level**			
Undergraduate students	386 (68.3%)	4.0 (3.0, 5.0)	0.388
Postgraduate students (master’s and doctoral students)	179 (31.7%)	4.0 (3.0, 5.0)	
**Employment status**			
Unemployed	361 (63.9%)	4.0 (3.0, 5.0)	0.419
Employed	204 (36.1%)	4.0 (3.0, 5.0)	
Paid employee	137 (24.2%)		
Public servant	38 (6.7%)		
Self-employed	29 (5.1%)		

* Shapiro–Wilk normality test. Statistically significant for *p* < 10^−3^. Mann–Whitney U test used.

**Table 2:** Final scores and classification obtained by the subjects when the HSQ was scored for migraine in the overall population: total sample: *N* = 565.

	*n* (% of Total Sample)
**Total score for migraine**	
0	4 (0.7%)
1	12 (2.1%)
2	117 (20.7%)
3	143 (25.3%)
4	128 (22.7%)
5	48 (8.5%)
6	70 (12.4%)
7	20 (3.5%)
8	23 (4.1%)
**Total score for migraine (summary statistics)**	
Mean (SD)	3.84 (1.71)
Median (IQR)	4.0 (3.0–5.0)
Shapiro–Wilk *p*-value *	<0.001
**Classification for migraine**	
No migraine	452 (80%)
Probable migraine	113 (20%)

* Shapiro–Wilk normality test.

**Table 3:** Relationship between basic demographic and migraine presence as determined by the HSQ-EV: total sample: *N* = 565.

	Number of Observations	Presence of Migraine		Pearson’s Chi-Squared Test
		Probable (*N* = 113)	No (*N* = 452)	
Demographic Characteristics		*n* (% of Row)	*n* (% of Row)	*p*-Value
**Sex**				
Male	98	15 (15.3%)	83 (84.7%)	0.201
Female	467	98 (21.0%)	369 (79.0%)	
**Marital status**				
Unmarried/divorced	520	98 (18.8%)	422 (81.2%)	0.020 *
Married	45	15 (33.3%)	30 (66.7%)	
**Educational level**				
Undergraduate students	386	71 (18.4%)	315 (81.6%)	0.161
Postgraduate students (master’s and doctoral students)	179	42 (23.5%)	137 (76.5%)	
**Employment status**				
Unemployed	361	63 (17.5%)	298 (82.5%)	0.044 *
Employed (paid employee, public servant, or self-employed)	204	50 (24.5%)	154 (75.5%)	

* Statistically significant for *p* < 0.05.

**Table 4:** Multivariate binary logistic regression analysis regarding variables associated with the presence of migraine.

		Multivariate Logistic Regression Analysis	
Variables	Category vs. Reference	OR (95% CI)	*p*-Value
Sex	Female vs. Male	1.68 (0.91–3.10)	0.100
Age	≥21 vs. <21 years old	1.86 (1.09–3.16)	0.022
Educational level	Undergraduate students vs. postgraduate students (master’s and doctoral students)	1.29 (0.73–2.26)	0.384
Marital status	Married vs. unmarried/divorced	1.59 (0.78–3.22)	0.202
Employment status	Employed (paid employee, public servant, or self-employed) vs. unemployed	1.26 (0.76–2.08)	0.374

The modeled probability was the presence of migraine ‘probably present’ versus ‘no’. The multivariate logistic regression model included the following covariates: sex, age group (≥21 vs. <21 years), marital status (married vs. unmarried/divorced), educational level (undergraduate vs. postgraduate), and employment status (employed vs. unemployed). These variables were selected based on clinical relevance and findings from univariate analyses. Abbreviations: CI, confidence interval; OR, odds ratio.

## Data Availability

Data available upon request due to restrictions. The data presented in the study are available upon request from the corresponding author due to personal data protection.

## Supplementary Materials

The following supporting information can be downloaded at: https://www.mdpi.com/article/10.3390/healthcare13141746/s1, Table S1: Headache screening questionnaire of the overall population: Total sample: *N* = 565.
