# Supplementary material for: Migraine Among University Students: Prevalence, Characteristics, and Sociodemographic Influences
Source: Healthcare (Basel). 2025 Jul 18;13(14):1746. doi: 10.3390/healthcare13141746 (PMC12294362; doi:10.3390/healthcare13141746)
Supplement: Supplementary file 1 [file healthcare-13-01746-s001.zip › healthcare-3691972-supplementary.pdf]

Supplementary Table S1. Headache screening questionnaire of the overall population: Total sample: N=565	
	n (% of total sample)
**Frequency of headache**	
**How often in your life have you had a headache?**	
0-4 times	228 (40.4%)
5-9 times	96 (17%)
≥10 times	241 (42.7%)
**How often would you describe those headache moments as a headache attack?**	
0-4 times	380 (67.3%)
5-9 times	118 (20.9%)
≥10 times	67 (11.9%)
**How many days per month do you have headaches?**	
<1 per month	270 (47.8%)
≥1 - <15 per month	282 (49.9%)
≥15 per month	13 (2.3%)
**Duration of headache**	
**How long does your headache last when you do not take any medication?**	
0-30 min	186 (32.9%)
30 min - 4h	315 (55.8%)
4h - 3 days	62 (11%)
3 - 7 days	2 (0.4%)
**Characteristics of headache**	
**What word would you use to describe your headache?**	
Tight or pressing feeling	354 (62.7%)
Pulsating feeling	111 (19.6%)
Other	63 (11.2%)
Burning or stabbing feeling	37 (6.5%)
**Is your headache one-sided or two-sided in nature?**	
Two-sided	292 (51.7%)
One-sided	273 (48.3%)
**What is the severity of your headache?**	
Mild	248 (43.9%)
Moderate	278 (49.2%)
Severe	37 (6.5%)
Very severe	2 (0.4%)
**Do daily activities (such as climbing stairs or walking) make your headache worse?**	
Yes	205 (36.3%)
No	360 (63.7%)
**Do you avoid daily activities when you have a headache?**	
Yes	260 (46%)
No	305 (54%)

Supplementary Table S1. Headache screening questionnaire of the overall population: Total sample: N=565

	**n (% of total sample)**
**Symptoms of headache**	
**During your headache, do you experience sensitivity to light?**	
Yes	224 (39.6%)
No	341 (60.4%)
**During your headache, do you experience sensitivity to sound?**	
Yes	303 (53.6%)
No	262 (46.4%)
**During your headache, do you experience nausea and/or vomiting?**	
Yes	140 (24.8%)
No	425 (75.2%)
**During your headache, you don't experience sensitivity to light/sound or, nausea or vomiting**	
Yes	93 (16.5%)
No	472 (83.5%)
**During your headache, do you experience other symptoms rather than sensitivity to light/sound or nausea or vomiting?**	
Yes	83 (14.7%)
No	482 (85.3%)
